# Interventional Radiotherapy in Gynecological Cancer

**DOI:** 10.3390/cancers15194804

**Published:** 2023-09-29

**Authors:** Angeles Rovirosa, Meritxell Arenas, Luca Tagliaferri

**Affiliations:** 1 Radiation Oncology Department, Hospital Clínic-Universitat de Barcelona, 08036 Barcelona, Spain; 2 Fonaments Clínics Department, Universitat de Barcelona, 08036 Barcelona, Spain; 3 Radiation Oncology Department, Hospital Sant Joan de Reus, Universitat Rovira Virgili, 43007 Tarragona, Spain; meritxell.arenas@salutsantjoan.ca; 4 U.O.C. Radioterapia Oncologica, Dipartimento di Diagnostica per Immagini, Radioterapia Oncologica ed Ematologia, Fondazione Policlinico Universitario “A. Gemelli”, IRCCS, 00168 Rome, Italy; luca.tagliaferri@policlinicogemelli.it

This special issue of “Cancers” explores unusual and very particular aspects of interventional radiotherapy (brachytherapy) in gynecological cancer.

To introduce the topic we will start with a short comment on the origin of brachytherapy, which is now called Interventional Radiotherapy. Brachytherapy involves the treatment of a tumor with radioactive sources introduced directly inside the tumor or through cavities using specific applicators for tumor access and treatment.

Radioactivity was discovered by Becquerel in 1896 and brachytherapy began with the discovery of radium by Marie Skolodowska-Curie in Paris in 1898. In 1901, Danlos and Block started using radium for lupus treatment, and in 1905 the use of brachytherapy spread to the United States. The first treatments with radium were for tumors of the skin and the cervix, and over time radium has been used in all the tumor sites. Systems for planning and dose administration were subsequently developed: the Stockholm system was developed in 1914, the Paris system in 1919 and the Manchester system in the 30s. In 1934, artificial radionuclides such as ^60^Co, ^81^Tantal, ^82^Au and ^192^Ir were discovered by Irene Curie and Frederick Joliot for use in clinical practice. All these treatments were performed manually with exposure of the health workers. In the decades of the 50s and 60s, new flexible and adaptable sources were discovered, including ^53^I, ^137^Cs, and ^192^Ir and different applicators were developed for each tumor site. The sources were of low-dose and median-dose rates. The most important aspects for health care personnel protection were the development of storage systems for the previously mentioned sources for transfer into the applicators by remote control. New guidelines were developed for Parys System the ^192^Ir By Chassagne, Pierquin and Dutreix. In the 90s, high-dose rate (HDR) sources and planning systems were developed with the inclusion of computerized tomography (CT) in the planning. At the beginning of the 21st century, applicators for CT planning were used by interventional Radiation Oncologist for HDR and pulsed dose rate (PDR) treatments and the manual charging of sources progressively disappeared. Different recommendations for treatment administration are published in different ICRU guidelines for intracavitary and interstitial brachytherapy, with the last ICRU 89 being specifically for cervical cancer. The most important innovations of the new interventional radiotherapy (HDR or PDR) compared to the old brachytherapy (linear sources) is the implementation of the possibility of intensity modulation in clinical practice, thanks to remote after loading machines with stepping source technology, and the image-guided approach [1,2,3].

Some trials and prospective studies have been developed and published demonstrating the relevance of brachytherapy in different tumor sites and can be found in 2 recently published brachytherapy books [4,5]. The present issue aimed to include different aspects in need of more precise study in order to obtain a better analysis for scientific, medical doctor and physicist awareness and for evolving brachytherapy in gynecological cancer.

Most of the modern applicators are very useful for adequately covering the tumor with the necessary dose. Nevertheless, in some cases commercial applicators are not able to adequately treat the tumors, leading to a consequent increase in disease relapse. In the last 10 years, case-specific 3D-printed applicators are progressively being manufactured and used in some centers. A wide narrative review written by Segedin et al. describes the different 3D-printed technology for use in cases in which manufactured applicators are not useful. The review by these authors shows that these 3D-printed applicators allow better dose coverage and an increase of the dose in the tumors, as well as better results in local control and less dose to organs at risk, including the vagina. Consequently, the use of this technology will increase in the next years [6].

At present, despite their evolution, planning systems have problems which have not been well resolved for use in daily practice, and in the present issue, we have included what will be needed in the future in order to increase the quality of planning systems and facilitate the work of physicians. Otal et al. summarize the possibilities offered nowadays by commercial TPSs, highlighting the absence of some useful tools that would notably improve the planning of magnetic resonance imaging (MRI)-based interstitial component cervical brachytherapy [7].

Analysis of quality assurance is necessary and should be included in clinical practice in order to ensure the safety of the treatments, which is reported by Soror et al. Quality assurance is aimed at standardizing the technical and clinical procedures involved in the treatment of patients, which could eventually decrease the source of uncertainties, whether they be technical source/equipment or clinically related. This review sheds light (from a clinical point of view) on some potential sources of uncertainties associated with the use of modern brachytherapy in the treatment of gynecological cancers. The authors nicely present problems and solutions during the selection of applicators, the use of imaging for image guided brachytherapy (IGBT), treatment planning and dose painting, as well as the role of in vivo dosimetry and recommend the use a quality assurance protocol [8].

Considering different tumor sites, the main indication and most common use of interventional brachytherapy is gynecological cancer, mainly postoperative endometrial cancer (PEC), followed by cervical cancer and then that of the vulva and also the vagina. Cervical cancer is most common in undeveloped areas but has a lower frequency in developed countries [9]. An ancient series by Baud J, including 451 patients treated with brachytherapy between 1919 and 1946, reported 5-year survivals of 58%, 43% and 10% and 0% for stages I, II, III and IV, respectively. Advances in external beam techniques, planning systems, volume definition using MRI and the use of interstitial brachytherapy, when necessary, in the Embrace I study showed a 72% overall survival and 92% local control at 5 years when considering all the stages [10]. At present, almost all relevant prospective studies in cervical cancer are from the Gynae GEC-ESTRO Working Group which, at present, is working hard on the reduction of toxicity in the Embrace II study and is now preparing the Embrace III study for launching and patient entry [11].

Endometrial cancer (EC) is the most common gynecological cancer in developed countries. The administration of exclusive brachytherapy after surgery in intermediate-high-risk EC offers vaginal relapse rates of up 2% in the PORTEC 2 trial. In the same way, in a study by Sorbe et al. external beam irradiation plus brachytherapy offered fewer vaginal relapses with no impact on survival [12,13]. At present, 3D-planning brachytherapy has not been well developed and analyzed in this setting, and there is wide heterogeneity in most of the treatment aspects. The last guidelines of the European Society of Gynecological Oncology, European Society for the Treatment of Radiation Oncology and European Society of Pathology (ESGO-ESTRO-ESP) the treatment recommendations were based on the agreement of experts and several publications. In the present issue, Markus et al. reports that there is no agreement in the different indications, doses, and treatment length in brachytherapy in 18 relevant centers in Europe [14]. A few years ago, the treatment results in local control and survival in PEC became a topic of interest, mainly after the analysis of biological markers for defining groups of risk for treatments. Recently, the publication of the results of biological markers from the PORTEC-1 and PORTEC-2, and the PORTEC-3 studies have shown different outcomes depending on the marker [15,16]. Considering brachytherapy, the most common fractionation schedules are 7Gyx3 and 5Gyx4. Zhang et al. showed that when doses of 6Gyx3 and 7.5Gyx2 are prescribed at 5 mm with an active source length of 2.5 cm, the incidence of complications is reduced, with no increase in relapses [17]. In a posterior work by the same authors, these fractionations were shown to be similar and the main aspect is that a lower number of fractions should offer greater patient comfort [18]. Data not yet published show no differences between the 2 schedules or in complications, relapses or dosimetric parameters.

Inoperable EC has been considered by several authors as a palliative treatment over the years. A retrospective study by the Gynae Group Europeen de Curietherapy working Group (GEC) of the ESTRO society (GEC-ESTRO) has shown that IGBT as exclusive treatment in Stage I inoperable EC, when indicated, and the association of EBRT + BT in stages I-III, provide benefits in the control of uterine disease with nice survival rates in patients that would die without these treatments [19,20]. Nonetheless, prospective studies are needed to establish relations between dose and volumes for increasing local control and survival.

Vaginal and vulvar cancer are in the phase of developing guidelines for treatment by different brachytherapy groups [21,22,23].

Another non-usual aspect is re-irradiation using brachytherapy. Patients presenting relapse after previous treatments are frequently unsuitable for other treatments. Bockel et al. reported good local control and survival and also moderate late complications in patients requiring re-irradiation for gynecological cancer in different tumor sites [24].

The sexuality of the patients undergoing different radiotherapy treatment modalities has not been well studied and needs further analysis. The use of vaginal dilators in these patients seems to reduce vaginal complications but is probably not the only aspect that should be taken into account after treatments [25,26].

Image-guided interventional radiotherapy in gynecological cancer is the most common in the clinical practice, and currently provides great benefits with very good results in local control and survival with few complications. At present, MRI should be used whenever possible, mainly when a tumor is present. Looking to the near future, among other aspects, the use of artificial intelligence may be very helpful in reducing treatment time in these patients [27]. The present issue presents the analysis of some special and unusual aspects of interventional radiotherapy gynecological cancer. New and interesting aspects will be shown in a new issue on Radiotherapy in Gynecological Cancer. Status of the art.
